# The epileptic blip syndrome

**DOI:** 10.1016/j.ebr.2024.100691

**Published:** 2024-06-26

**Authors:** Edgar Matringe, Juan R. Vidal, Marcela Perrone-Bertolotti, Laurent Vercueil

**Affiliations:** a Univ. Grenoble Alpes, Univ. Savoie Mont Blanc, CNRS, LPNC, 38000 Grenoble, France; b Lyon Catholic University, UR CONFLUENCE : Sciences et Humanités (EA 1598), 69002 Lyon, France; c Service des Explorations Fonctionnelles du Système Nerveux, CHU Grenoble Alpes, 38000 Grenoble, France

**Keywords:** Blip syndrome, Epilepsy, Generalized spike-wave bursts, Consciousness stream

## Abstract

We report on the case of an adolescent with juvenile myoclonic epilepsy exhibiting compulsory sporadic voluntary movement. These movements entailed the deliberate act of touching her forehead with her hand and were triggered by a short and indefinable cephalic sensation. Upon inquiry regarding the nature of this movement, the patient reported a sudden perception of a peculiar event localized “inside her head”. In the course of a prolonged video-electroencephalographic recording, it appeared that the movements consistently followed the occurrence of diffuse Generalized Spike-and-Wave Bursts (GSWBs), lasting one to three seconds. This observation suggests that the impact of the GSWBs on the patient’s stream of consciousness could be detected by the patient herself, who attributed it to an internal sensation, like a “blip on a screen”. This clinical observation echoes the “blip syndrome”, as described by the neurologist James W. Lance thirty years ago. These findings give some support that “blips” could be of an epileptic origin and lend weight to the notion that some patients could perceive their own EEG discharges. Moreover, this observation should be added to the perennial debate on the cognitive impact of intercritical discharges.

## Highlights

• The “blip syndrome” could be of an epileptic origin.
• Brief generalized spike-wave bursts can impact the stream of consciousness.
• Diverse forms of brief generalized spike-wave bursts may exist.
• A spectrum of potential manifestations may be associated with these bursts.

## 1 Introduction

Electrophysiological recording through electroencephalogram (EEG) highlights interictal epileptiform bursts (IEBs). By definition, IEBs manifest as abrupt and transient graphic elements, with a duration ranging from 20 ms to 3 s. IEBs are more frequently observed during an eyes-closed drowsy state and during sleep [1]. The prevalence of IEBs in the population without epilepsy lies between 0.4 % and 12.3 % [2]. Within the population with epilepsy, the frequency of IEBs is higher, particularly in instances of early diagnosis and higher seizure rate [3]. The classification of IEBs is based on the morphological features and the extent of the bursts. Despite usually being defined as devoid of visible clinical manifestations, the impact of IEBs remains controversial, with no consensus on their effects. Recent investigations in cognitive neurosciences and neuropsychology have underscored the potential impact of IEBs on cognition. Aarts and colleagues have suggested that IEBs can involve direct “Transitory Cognitive Impairment” in short-term memory tasks [4]. Additionally, like epileptic seizures [5], several studies have established a link between the frequency of IEB and subsequent effects on global cognitive processes, including processing speed and attentional stability [6], [7], [8], [9], [10]. Significant considerations are discussed concerning the characterization of potential cognitive impacts attributed to IEBs, including the critical aspects of rhythmicity, topographical distribution, lateralization, and duration of these bursts.

In the case of focal IEBs, distinctive electrophysiological signatures arise from various cortical areas [11]. Conversely, Generalized Spike-Wave Bursts (GSWBs) have traditionally been regarded as a unique phenomenon, so far. Notably, at the group level, EEG-fMRI studies have revealed blood-oxygen-level-dependent modifications occurring during the course of GSWBs EEG recording. These modifications specifically involved cortical regions, such as the precuneus/posterior cingulate, lateral parietal, and frontal cortices [12], [13], [14], [15]. Some of these regions belong to the so-called default mode network [16]. Notably, such modifications were also observed in typical absence seizures [17], which are epileptic seizures characterized by longer generalized spike-wave discharges at 3 Hz, with a duration of approximately 10 s [18], [19]. However, unlike interictal GSWBs, absence seizures are typically characterized by sudden, brief episodes of staring and unresponsiveness. They are considered the most “pure” example of impaired consciousness in epilepsy [20]. Given the attributes of brief interictal GSWBs, such as their prevalence during waking activity, increased frequency during periods of rest, and BOLD modifications similar to those observed in absence seizures, we hypothesized that these brief GSWBs may impact the perception of continuous consciousness stream.

In revisiting the literature, James W. Lance’s delineation of the “blip syndrome” resonates with our current theoretical framework [21]. Lance characterized the blip syndrome as a *“*momentary sensation of impending loss of consciousness,” often manifesting when the patients were “relaxing, reading, listening to music, or watching television. One mentioned that she had an attack while lying on bed and that another had awakened her from sleep”. Blip syndrome patients were presenting “without any obvious cardiac, cerebral vascular or epileptic basis”. Lance proposed that these very brief episodes could be “quasiepileptic phenomenon such as déjà vu and night starts and seem to have a benign prognosis”. Notably, patients experiencing the blip syndrome may report multiple episodes per day. In Lance’s initial case series encompassing 12 patients with the blip syndrome, investigations in both neurological and cardiac examinations were negative. Building upon the descriptive elements presented by James W. Lance in the literature, we propose three distinct general criteria to characterize the phenomenological description of the blip syndrome: 1) Negative symptoms would be associated with blips and would commonly be described as an impending sensation of losing consciousness; 2) Blips would be exceedingly brief, perceived to last less than a second; 3) Blips would be phenomenologically localized within the interior of the head.

In summary, parallels emerge between the clinical characterization of blips and the neurophysiological signature of GSWBs. Both phenomena share brevity in duration. Furthermore, they exhibit a propensity for recurrent episodes throughout the day, with an increased frequency during periods of rest. In this case study report, we took the opportunity to present a young person with epilepsy experiencing the “blip syndrome” temporally synchronized with concise generalized spike-and-wave bursts on EEG. This observation underscores that a blip could be of an epileptic origin.

## 2 Case report

A 15-year-old patient diagnosed with juvenile myoclonic epilepsy exhibited an intriguing clinical manifestation — a compulsory sporadic voluntary movement involving the touching of her forehead with her hand, the movement being triggered by an indefinable, very brief cephalic sensation. The patient described feeling a peculiar event “inside her head” as the catalyst for the movements. No significant past medical history or neurodevelopmental conditions were evidenced, and the family history for neurological disorders was negative, except for her mother who also suffered from epilepsy. No genetic testing was performed. The patient did not experience any other types of epileptic seizures, such as eyelid myoclonia nor absence seizures. Additionally, the patient was on valproic acid as an anti-seizure medication, and no side effects were reported.

**Fig. 1:**
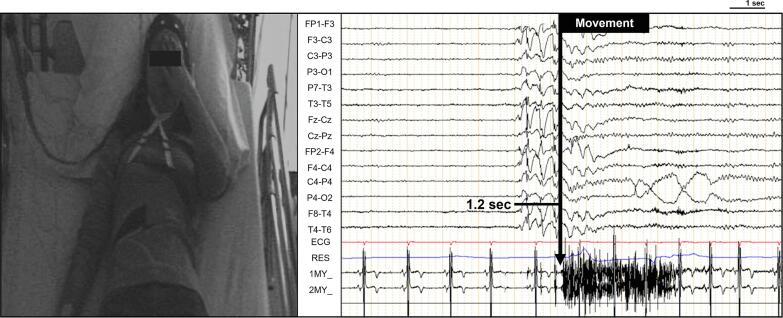
Captured video-EEG/EMG of a temporal relationship between the patient’s auto-reported blip-like sensation, the voluntary movement, and the occurrence of a brief GSWB. *Note: The voluntary movement starts 1.2 s after the initiation of the GSWB (2 s). Upon inquiry about these movements, the patient self-reported a peculiar sensation localized behind her forehead, highlighting the intricate relationship between the phenomenological experience and a neurophysiological GSWB.*

Prolonged video-electroencephalogram recordings revealed a precise temporal association between the sensations and the voluntary movements with diffuse generalized spike-wave bursts, lasting no longer than one to two seconds (Fig. 1). Following these sensations, the patient was able to describe a detailed account of her ictal semiology. She characterized the experience as peculiar sensations and metaphorically likening it to a reasoning box — her own head — and attributing it to an internal sensation “like a blip on a screen”. Importantly, no sensorial components were associated with the sensations. During the extended video-EEG recording, a total of thirty GSWBs were registered. Among these, eighteen were asymptomatic, six presented with myoclonus and nine were accompanied by blip-like sensation, all temporally synchronized with short GSWBs (Table 1). Six blip-like sensations were followed by a voluntary movement — touching her forehead with her hand — immediately after the occurrence of GSWBs (*M*: 1.1 s, *SD*: 0.30), as shown in several instances in Video 1, and three were only verbally reported by the patient. Additionally, three of the blip-like sensations were associated with myoclonus.

**Table 1:** Descriptive summary of GSWB recording during patient’s video-EEG (140 min).

GSWBs (Number)	Asymptomatic (18)		Myoclonus (6)		Blip-like Sensations (9)		Total (30)	
	*M*	*SD*	*M*	*SD*	*M*	*SD*	*M*	*SD*
Duration (sec)	1.58	0.55	1.75	0.40	2.06	0.46	1.73	0.55

## 3 Discussion

We present a case study detailing a patient whose experiences involve a peculiar sensation, metaphorically akin to “a blip on the screen”, intricately synchronized with the occurrence of GSWBs. Notably, these events may be accompanied by atypical voluntary gestures involving the patient touching her forehead. Our observation suggests that the impact of GSWBs in the stream of consciousness is discernible to the patient.

Drawing from the patient’s detailed neurophenomenological account, we assume that the sensations are distinct from brief attentional disturbances. An unwanted mobilization of attentional resources is generally triggered from exteroceptive or interoceptive information. The described manifestations do not manifest as weakness, dizziness, tingling, tickling, or itching in any part of the body. Instead, they resemble an “almost black-out”, a “mild black-out” or a forced “mindblanking” [22], [23]. Crucially, these phenomena are intra-psychic and localized by the patient to the area “behind her forehead”. Given the absence of sensations more precisely defined than an “impending loss of consciousness”, we propose that these experiences do not align with classical interoception, nor with exteroception involving auditory or visual sensory stimulation. The patient does not report any pain, nociceptive cephalic sensations, or headaches. Furthermore, similarly to the description of the blip syndrome, the patient described these events as exceptionally brief and mentioned that they tend to occur more frequently at rest. This case report suggests the intriguing hypothesis that the blip syndrome might be a phenomenon of the brain’s detection of generalized spike-wave bursts. If validated, such a phenomenon might be highly surprising considering the brain's general insensitivity to its own pain. It would also introduce a novel dimension to the comprehension of the intricate interplay between neural activity and the perception of continuous conscious experience.

What signaled the phenomenon to the investigators was the movement of the hand raised to the forehead. Time analysis showed that the initiation of the movement (EMG onset) occurred after GSWB onset, with a delay of approximately one second. The patient revealed the voluntary nature of the movement, which was assumed to be a behavioral response to the cephalic sensation. It was therefore not strictly speaking a matter of ictal automatism. Moreover, this temporal pattern excludes other rare photosensitive epilepsies involving handwaving, such as in sunflower syndrome or in eyelid myoclonia with absence (Jeavons syndrome) [24].

The ictal nature of the gestures observed in sunflower syndrome has been debated [24], but a recent study suggests that the movements shortly follow, and do not precede, the start of epileptiform discharges [25]. However, the gesture in sunflower epilepsy is typically a highly stereotyped hand-waving motion, whereas in this case, the patient touched her forehead only once after the discharge starts. Additionally, the patient did not specifically orient towards a natural or artificial light source, which is characteristic of sunflower syndrome. The movements reported in this case study were not systematically present when the patient experienced a blip-like sensation and were not associated with any specific relief, such as agitation or stress, before, during, or after the sensation.

The neurophenomenological depiction of the “epileptic blip syndrome” offers valuable theoretical insights, suggesting its emergence from an abrupt and transient disruption in the continuity of consciousness stream. On one hand, distinguishing elements, such as the notably shorter duration of blips (phenomenologically reported as less than one second), the distinctly different clinical semiology of blips-like episodes, and the lack of typical absence seizures in this patient lead us to propose that epileptic blips differ from absence seizures. On the other hand, the semiology of blips – an impending sensation of losing consciousness – echoes the classical description of absence seizures [20], which are non-motor seizures often described as abrupt and transient loss of consciousness. Furthermore, the pathognomonic generalized spike-wave discharges between 2 and5 Hz resemble the discharges observed in blips, but the blips are shorter in duration. Thus, it is conjectured that these blips may, at the very least, represent a fragmentary aspect of absence seizures. This nuanced understanding underscores the complexity of epileptic manifestations and highlights the need for further exploration to delineate the unique features and underlying mechanisms of the epileptic blip syndrome.

In exploring the blip syndrome through the model of epilepsy, we propose that short GSWBs may be classified into distinct categories, some may impact the perception of continuity in the consciousness stream, while others remain clinically “silent”. This observed disruption in the stream of consciousness holds promise for advancing our understanding of the underlying neurophenomenological mechanisms of consciousness. While the intensity of GSWBs may constitute a significant factor influencing the impact on consciousness experience, we suggest a different perspective. Similar electrophysiological signatures of GSWBs on EEG may, in fact, generate a spectrum of clinical semiology, each engaging distinct neural network. These variations may rely on factors such as source localizations, interactions, and propagations of the bursts. From a global perspective, we hypothesize that GSWBs may engage cortico-subcortical networks involved in the maintenance of the stream of consciousness. If validated, this diversity of GSWBs could provide novel insights into the variability of the results observed in fMRI/EEG research when considering epileptiform GSWBs as a singular phenomenon [12], [13], [14], [15]. Specifically, during epileptic blips, a substantial deactivation of neural networks supporting the stream of consciousness may be implicated [18]. To deepen our understanding, future investigations should meticulously explore the prevalence of the blip syndrome within specific populations with epilepsy, particularly in populations more susceptible to GSWBs occurrence. Such studies promise valuable insights into the nuanced interplay between GSWBs and consciousness, shedding light on the varied manifestations and potential network involvement associated with this intriguing neurophenomenological phenomenon.

## 4 Conclusions

In this case study, we reported the occurrence of compulsory sporadic voluntary gestures triggered by cephalic sensations, time-locked with brief generalized spike-and-wave bursts. The description of these sensations, closely echoes the characteristics of the blip syndrome, as initially introduced by James W. Lance thirty years ago [22]. Under the name of “epileptic blip syndrome” and for the first time to our knowledge, we propose that a blip could be of an epileptic origin. To define the general features of epileptic blip syndrome for the purposes of further studies, we propose four criteria based on the literature and this case report: epileptic blips would be 1) negative symptoms, often described as an impending sensation of losing consciousness; 2) exceedingly brief, perceived as lasting less than a second; 3) phenomenologically localized within the interior of the head; 4) strictly time-locked with the occurrence of short generalized spike-and-wave bursts on EEG recordings. This observation also has important theoretical value, as it suggests that subjects can occasionally perceive their own EEG discharges. Moreover, it revives the controversial debate on the cognitive impact of intercritical discharges.

## Ethical statement

The patient signed an informed consent for usage of data (institutional ethics committee approval number **CERGA-Avis-2024-08**).

## Declaration of Generative AI and AI-assisted technologies in the writing process

During the preparation of this work the authors used [ChatGPT-4, OpenAI, Large language model] in order to improve readability and language. After using this tool/service, the authors reviewed and edited the content as needed and take full responsibility for the content of the publication.

## CRediT authorship contribution statement

**Edgar Matringe:** Writing – review & editing, Writing – original draft, Visualization, Project administration, Formal analysis, Conceptualization. **Juan R. Vidal:** Writing – review & editing, Validation, Supervision. **Marcela Perrone-Bertolotti:** Writing – review & editing, Validation. **Laurent Vercueil:** Writing – review & editing, Writing – original draft, Validation, Resources, Project administration, Investigation, Conceptualization.

## Declaration of competing interest

The authors declare that they have no known competing financial interests or personal relationships that could have appeared to influence the work reported in this paper.

## Appendix A Supplementary data

Supplementary video 1 Video-EEG/EMG recordings of blip-like sensations time-locked to generalized spike-wave bursts, followed by voluntary gestures.
